# Mechanism of piR-DQ590027/MIR17HG regulating the permeability of glioma conditioned normal BBB

**DOI:** 10.1186/s13046-018-0886-0

**Published:** 2018-10-11

**Authors:** Xue Leng, Jun Ma, Yunhui Liu, Shuyuan Shen, Hai Yu, Jian Zheng, Xiaobai Liu, Libo Liu, Jiajia Chen, Lini Zhao, Xuelei Ruan, Yixue Xue

**Affiliations:** 1 Department of Neurobiology, College of Basic Medicine, China Medical University, Shenyang, 110122 People’s Republic of China; 2 Key Laboratory of Cell Biology, Ministry of Public Health of China, and Key Laboratory of Medical Cell Biology, Ministry of Education of China, China Medical University, Shenyang, 110122 People’s Republic of China; 3 Department of Neurosurgery, Shengjing Hospital of China Medical University, Shenyang, 110004 People’s Republic of China; 4 Liaoning Research Center for Clinical Medicine in Nervous System Disease, Shenyang, 110004 People’s Republic of China; 5 Key Laboratory of Neuro-oncology in Liaoning Province, Shenyang, 110004 People’s Republic of China

**Keywords:** piR-DQ590027, MIR17HG, miR-153, miR-377, FOXR2, Blood-brain barrier

## Abstract

**Background:**

The blood-brain barrier (BBB) strongly restricts the entry of anti-glioma drugs into tumor tissues and thus decreases chemotherapy efficacy. Malignant gliomas are highly invasive tumours that use the perivascular space for invasion and co-opt existing vessels as satellite tumor form. Because regulation of the effect of noncoding RNA on BBB function is attracting growing attention, we investigated the effects of noncoding RNA on the permeability of glioma conditioned normal BBB and the mechanism involved using PIWI-associated RNA piR-DQ590027 as a starting point.

**Methods:**

The mRNA levels of MIR17HG, miR-153, miR-377, ZO-1, occludin, and claudin-5 were determined using real-time PCR. Transient cell transfection was performed using Lipofectamine 3000 reagent. TEER and HRP flux were applied to measure the permeability of glioma conditioned normal BBB. Western blotting and immunofluorescence assays were used to measure ZO-1, occludin, and claudin-5 levels. Reporter vector construction and a luciferase reporter assay were performed to detect the binding sites of MIR17HG and piR-DQ590027, MIR17HG and miR-153 (miR-377), and FOXR2 and miR-153 (miR-377). RNA immunoprecipitation was used to test the interaction between miR-153 (miR-377) and its target proteins. Chromatin immunoprecipitation was performed to detect the interaction between the transcription factor FOXR2 and ZO-1, occludin, and claudin-5.

**Results:**

piR-DQ590027 was expressed at low levels in glioma-conditioned ECs (GECs) of the in vitro glioma conditioned normal BBB model. Overexpression of piR-DQ590027 down-regulated the expressions of ZO-1, occludin, and claudin-5 and increased the permeability of glioma conditioned normal BBB. MIR17HG had high expression in GECs but miR-153 and miR-377 had low expression. piR-DQ590027 bound to and negatively regulated MIR17HG. FOXR2 was a downstream target of miR-153 and miR-377; MIR17HG bound separately to miR-153 and miR-377 and negatively regulated their ability to mediate FOXR2 expression. FOXR2 associated with the promoter regions of ZO-1, occludin, and claudin-5 in GECs to promote their transcription.

**Conclusion:**

The piR-DQ590027/MIR17HG/miR-153 (miR-377)/FOXR2 pathway plays an important role in regulating glioma conditioned normal BBB permeability and provides a new target for the comprehensive treatment of glioma.

**Electronic supplementary material:**

The online version of this article (10.1186/s13046-018-0886-0) contains supplementary material, which is available to authorized users.

## Background

Most glioma is a primary intracerebral tumor with high lethality [1]. The current treatment approach includes surgery, chemotherapy, radiotherapy, and molecular targeted therapy, which itself involves chemotherapy [2]. Glioma cells invade along the existing normal capillaries in brain. Normal capillary endothelial cells function as the blood-brain barrier (BBB) that limits penetration of chemicals into the brain [3]. Though the features of blood-tumor barrier (BTB) in tumor tissues are different from BBB, it still limits the transport of macromolecular chemotherapeutics into glioma tissues [4] and severely reduces the efficacy of these drugs. A glioma conditioned normal BBB model was developed to simulate the tumor conditions to a degree, and to further explore the mechanism of noncoding RNAs regulating the permeability to seek opportunities for glioma treatment.

PIWI proteins are a subfamily of argonaute proteins. PIWI-associated RNA (piRNA) is a kind of noncoding RNA with a length of 20 to 33 nucleotides that exerts its biological functions by interacting with PIWI proteins. The initial work showed that piRNA was involved in generating germ cells and maintaining the structural stability of relevant cytogenes [5]. Recent studies have suggested that piRNA is associated with the occurrence and development of cancer. For example, piR-651 expression is increased in gastric, colon, lung, and breast cancer [6], whereas piR-823 has low expression in gastric cancer tissues [7]. The effects of piR-DQ590027 on the permeability of glioma conditioned normal BBB have not yet been reported.

Long noncoding RNA (lncRNA), another type of noncoding RNA, has a transcript length of over 200 nucleotides and plays vital roles in cancer biology. Some studies have reported that lncRNA TUG1 and MALAT1 help to regulate BTB permeability [8, 9]. One type of lncRNA is the miR-17-92a cluster host gene (MIR17HG). MIR17HG participates in tumor occurrence and development and affects the efficacy of neoadjuvant chemotherapy for rectal cancer, as well as the evaluation of treatment response [10]. The rs4824505 allele and AC haplotype of the rs4824505/rs7336610 form of the MIR17HG gene are positively correlated with breast cancer progression risk [11]. Using piRNABank and piRNA predictor bioinformatic software, we predicted the presence of a binding site for piR-DQ590027 in the base sequence of MIR17HG. Nonetheless, the impact of piR-DQ590027 on the permeability of glioma conditioned normal BBB via MIR17HG regulation is unclear.

miR-153 has low expression in tumor tissues and cell lines of pancreatic cancer, melanoma, and glioma, regulates such biological behaviors as proliferation, invasion, and apoptosis of these tumors, and acts as a tumor-suppressive factor [12–16]. miR-153 inhibits the cell proliferation of triple-negative breast cancer and glioblastoma multiforme [15, 16]. miR-377 has low expression in tumor tissues of esophageal cancer and small cell lung cancer [17, 18]. miR-377 suppresses human glioblastoma proliferation and invasion by targeted-regulation of specific protein 1 (Sp1) [19]. Additionally, miR-377 silencing promotes the growth and migration of squamous cell carcinoma [20], indicating that miR-377 plays a role as a tumor-suppressive factor in the above tumors. According to bioinformatics software prediction (starBase 2.0), MIR17HG contains binding sites for miR-153 and miR-377, suggesting that MIR17HG may regulate the permeability of glioma conditioned normal BBB by adjusting the functions of these miRNAs.

FOXR2 is a member of the forkhead box transcription factor family and highly expressed in liver, breast, lung, and prostate cancer [21–24]. In addition, FOXR2 promotes the proliferation and migration of colorectal carcinoma and acts as a carcinogen [25]. FOXR2 is highly expressed in medulloblastoma and also functions as a carcinogen [26], but the role of FOXR2 in regulating the permeability of glioma conditioned normal BBB has not yet been reported. Bioinformatics software prediction (TargetScan) showed binding sites for miR-153 and miR-377 in the 3’UTR of FOXR2 mRNA, indicating that miR-377 and miR-153 may regulate the permeability glioma conditioned normal BBB by adjusting the expression and function of FOXR2.

In this study, we investigated the expression of piR-DQ590027, MIR17HG, miR-153, miR377, and FOXR2 in glioma-conditioned ECs (GECs) and then further explored the possible regulatory relationships among the above factors and their mechanisms of action on the permeability of glioma conditioned normal BBB. This study aimed to reveal how noncoding RNA regulates the permeability of glioma conditioned normal BBB and provide new possibilities for antitumor drugs.

## Methods

### Cell lines and cultures

The immortal human brain EC line hCMEC/D3 was kindly provided by Dr. Couraud (Cochin Institute, Paris, France). Cells were cultured in culture flasks coated with Cultrex murine collagen I (R&D Systems, Minneapolis, MN) at 150 mg/mL. Cells were maintained in endothelial basal medium (EBM-2) (Lonza, Walkersville, MD, USA), containing 5% fetal bovine serum (FBS) “Gold” (PAA Laboratories, Pasching,Austria), 1% penicillin-streptomycin (Life Technologies, Paisley,UK), 1.4 mmol/L hydrocortisone (Sigma-Aldrich, St Louis, MO,USA), 1% chemically defined lipid concentrate (Life Technologies,Paisley, UK), 5 mg/mL ascorbic acid (Sigma-Aldrich), 10 mmol/LHEPES (PAA Laboratories), and 1 ng/mL human basic fibroblastgrowth factor (bFGF) (Sigma-Aldrich). Endothelial cells were limited from 30 to 40 passages. Human astrocytes were obtained from Scienell Research Laboratories (Carlsbad, CA, USA) and cultured in astrocyte medium RPMI-1640 (GIBCO, Carlsbad, CA, USA). The human glioma U87MG and human embryonic kidney 293 T (HEK293T) cell lines were purchased from the Cell Resource Center of Shanghai Institutes for Biological Sciences (Shanghai, China) and cultured with Dulbecco’s modified Eagle’s high glucose medium containing 10% fetal calf serum, 100 U/mL penicillin, and 100 μg/mL streptomycin (Life Technologies, Paisley, UK). Cells were maintained at 37 °C in a humidified incubator with 5% CO_2_ and refreshed medium every 48 h. All cell lines were examined with MycoGuard™ Mycoplasma PCR Detection equipment, and no mycoplasma contamination was found.

### Establishment of in vitro BBB and glioma-conditioned normal BBB model

The in vitro BBB and glioma-conditioned normal BBB model were established as described previously [8]. An in vitro BBB model or glioma-conditioned normal BBB model was created in a Transwell system. Briefly, human astrocytes or U87 cells were seeded into the lower chamber of 6-well transwell inserts at a density of 2 × 10^4^ cells per well. After culturing human astrocytes or U87 cells for 2 days, hCMEC/D3 were seeded on the upper chamber of transwell inserts pre-coated with Cultrex Rat Collagen I (R&D Systems) at a density of 2 × 10^5^ per well. Both lower and upper chamber were maintained with prepared endothelial basal medium 2 (EBM-2), and medium was refreshed every 2 days. Then the models were established successfully after co-culturing for 4 days. “ECs” represents co-cultured ECs (hCMEC/D3) with human astrocytes; “GECs” was glioma-conditioned ECs, which represents co-cultured ECs (hCMEC/D3) with glioma cells. In the subsequent experiments, either un-transfected (“Control”) or transfected cells were GECs, unless otherwise indicated.

### Real-time PCR assay

Total cell RNA was extracted using Trizol reagent (Life Technologies). A high-performance cDNA Reverse Transcription Kit (or TaqMan MicroRNA Reverse Transcription Kit) was used for lncRNA and mRNA (or miRNA) reverse transcription (Applied Biosystems, Foster City, CA). RNA concentration and mass were determined using a NanoDrop spectrophotometer (ND-100, Thermo Fisher Scientific, Waltham, MA). The levels of MIR17HG, FOXR2, ZO-1, occludin, and claudin-5 were measured using the single-step SYBR PrimeScript RT-PCR Kit (Perfect Real-Time; Takara Bio Inc., Kusatsu, Japan). Glyceraldehyde 3-phosphate dehydrogenase (GAPDH) was used as an endogenous control. The levels of piR-DQ590027 and miR-153 (miR-377) were detected by a TaqMan MicroRNA Reverse Transcription Kit and TaqMan Universal Master Mix II (Applied Biosystems). U6 was used as an endogenous control. The fold change calculation used the relative quantification (2^-ΔΔ^Ct) method. Primers and probes are shown in Table 1.

**Table 1:** Primers and probes used for RT-qPCR

Primer or Probe	Gene	Sequence (5′- > 3′) or Assay ID
Primer	MIR17HG	F:TCAGGAGTTCGAGACCAACC
		R:TGCCTCAGCCTCCAGAGTAG
	FOXR2	F:CTGTGAACCCAATCTGTGGA
		R:GGCTGAGGGAAAGGAGAAAT
	GAPDH	F: ACAGTCAGCCGCATCTTCTT
		R: GCCCAATACGACCAAATCC
Probe	MiR-153	rs180704631
	MiR-377	rs774685804
	U6	001973(Applied biosystems)

### Cell transfection

Transient cell transfection was performed using Lipofectamine 3000 reagent (Invitrogen, Carlsbad, CA) according to the manufacturer’s protocol. Short hairpin RNA constructs for the human MIR17HG and FOXR2 genes were constructed in a pGPU6/GFP/Neo vector shMIR17HG, shFOXR2; GenePharma, Shanghai, China. The empty vector was used as a negative control (NC) [NC (−) and FOXR2 (−) NC)]. The human FOXR2 gene coding sequence was connected to the pIRES-EGFP vector [FOXR2 (+)] (GeneScript, Piscataway, NJ), and its null vector was used as an NC [FOXR2 (+) NC]. The ECs were inoculated in 24-well plates and were transfected with Opti-MEM I and Lipofectamine LTX reagents (Life Technologies Corp., Carlsbad, CA) according to the manufacturer’s instructions. Stably transfected cell lines were selected using G418. G418-resistant clones were obtained after 4 weeks. Cells were collected 48 h after transfection. The sequences of shMIR17HG, shFOXR2, and shNC are shown in Table 2.

**Table 2:** Sequences of shRNA template

Gene		Sequence(5′- > 3′)
MIR17HG	Sence	CACCGGTGGCCTGCTATTTCCTTCATTCAAGAGATGAAGGAAATAGCAGGCCACCTTTTTTG
	Antisence	GATCCAAAAAAGGTGGCCTGCTATTTCCTTCATCTCTTGAATGAAGGAAATAGCAGGCCACC
FOXR2	Sence	CACCGGATCTGACAAACATTTCTCCTTCAAGAGAGGAGAAATGTTTGTCAGATCCTTTTTTG
	Antisence	GATCCAAAAAAGGATCTGACAAACATTTCTCCTCTCTTGAAGGAGAAATGTTTGTCAGATCC
NC	Sence	CACCGTTCTCCGAACGTGTCACGTTTCAAGAGAACGTGACACGTTCGGAGAATTTTTTG
	Antisence	GATCCAAAAAATTCTCCGAACGTGTCACGTTCTCTTGAAACGTGACACGTTCGGAGAAC

### TEER assays

TEER values were measured in an in vitro glioma-conditioned normal BBB model using a Millicell-ERS instrument (Millipore, Billerica, MA). In short, U87 cells were inoculated in 6-well plates with 2 × 10^4^ cells per well and cultured for 2 days. The co-culture inserts were placed at room temperature for 30 min and then the TEER values were measured immediately after the culture medium was replaced. The background resistance was subtracted before the final resistance was calculated. The Transwell insert surface area is expressed as Ω•cm^2^.

### Horseradish peroxidase flux assays

After the in vitro glioma-conditioned normal BBB model was established, 1 mL culture medium containing 10 mg/mL HRP (0.5 mM, Sigma-Aldrich) was added into the upper system and 2 mL of culture medium was added to the well. Then, 5 μL of culture medium was collected from the lower compartment after incubation at 37 °C for 1 h. Samples were subjected to colorimetric analysis using tetramethylbenzidine. Absorbance was measured at 370 nm with a spectrophotometer. The final HRP flux was expressed as pmol passed per cm^2^ surface area per hour.

### Western blot

Total proteins were extracted with RIPA buffer (Beyotime Institute of Biotechnology, Jiangsu, China) supplemented with protease inhibitors (10 mg/mL aprotinin, 10 mg/mL phenyl-methylsulfonyl fluoride [PMSF], and 50 mM sodium orthovanadate) and centrifuged at 17,000×g for 30 min at 4 °C. Then, a bicinchoninic acid protein assay kit (Beyotime Institute of Biotechnology) was used to determine the protein concentration of the supernatant. Equal amounts of these total proteins (50 mg) were separated using SDS-PAGE and electrically transferred onto a polyvinylidenedifluoride membrane (Millipore, Shanghai, China). After non-specific binding was blocked with 5% nonfat milk in Tris-buffered saline/Tween 20 (TBST) at room temperature for 2 h, membranes were incubated with primary antibodies as follows: GAPDH (1:10,000; Proteintech, Rosemont, IL), ZO-1 (1:300; Life Technologies Corp.), occludin (1:1000; Proteintech), and claudin-5 (1:30; Life Technologies Corp.). Then, membranes were incubated with HRP-conjugated secondary antibody diluted at 1:5000 at room temperature for 2 h. After washing three times with TBST, protein blots were visualized using an enhanced chemiluminescence kit (ECL) (Santa Cruz Biotechnology, Dallas, TX) and detected with an ECL Detection System (Thermo Scientific, Beijing, China). Protein bands were then scanned using Chemi Imager 5500 V2.03 software, and integrated light density values were calculated using Fluor Chen 2.0 software, with GAPDH as an internal control.

### Immunofluorescence assays

Cells were fixed with 4% paraformaldehyde for 20 min at room temperature and permeated in PBS containing 0.2% Triton X-100 for 10 min (ZO-1 and claudin-5) or fixed with methanol for 10 min at − 20 °C (occludin), followed by incubation in 5% BSA blocking buffer for 2 h at room temperature. Then, cells were incubated with primary antibodies against ZO-1 (1:50; Life Technologies), occludin (1:50; Abcam, Cambridge, UK), and claudin-5 (1:50; Life Technologies) overnight at 4 °C. After washing three times with PBS/Tween 20, cells were incubated with AlexaFluor-555-labeled goat anti-mouse IgG or anti-rabbit IgG secondary antibody (1:500; Beyotime Institute of Biotechnology) for 2 h at room temperature. The nuclei were then counterstained with 0.5 mg/mL DAPI for 5 min. Staining was visualized using confocal microscopy (confocal microscopy parameters: gain value, 2; gamma value, 1; DAPI laser strength, 79%; Alexa Fluor, 68%).

### Reporter vector construction and luciferase reporter assay

pmirGLO dual-luciferase carrier (Promega, Madison, WI) was cloned into the downstream region of the pmirGLO dual-luciferase carrier (MIR17HG-piR-Wt), which was determined by the amplified MIR17HG gene. Similarly, pmirGLO dual-luciferase vectors were constructed to contain the putative miR-377 binding sequence in the MIR17HG gene (MIR17HG-miR-Wt) or its respective mutant sequence (MIR17HG-miR-Mut), the putative miR-153 binding sequence in the MIR17HG gene (MIR17HG-miR-Mut1 and MIR17HG-miR-Mut2) or its respective mutant sequence, and the FOXR2 3’UTR (FOXR2-miR-Wt) or its respective mutant sequences (FOXR2-miR-Mut). Lipofectamine 3000 was used to transfect HEK293T cells with pmirGLO dual-luciferase vector (wild-type or mutant fragment) and pre-piR-DQ590027 or pre-piR-NC. The transfection method and luciferase activity were determined by the Promega Fluorescein Enzyme Activity System after 48 h of transfection.

### RNA immunoprecipitation assay

RNA immunoprecipitation assays were performed using the Magna RNA binding protein immunoprecipitation kit (Millipore). In short, cells were lysed in complete RNA lysis buffer, and the cell lysate was incubated with human anti-Ago2 antibody (Millipore) and NC mouse IgG (Millipore). Anti-SNRNP70 was used as a positive control. Quantitative real-time PCR was performed after isolation and purification of immunoprecipitated RNA.

### Chromatin immunoprecipitation assay

Chromatin immunoprecipitation was determined by Cell Signaling Technology (Danvers, MA) using a simple chip enzyme. In short, the cells were cross-linked in EBM-2 containing 1% of formaldehyde for 10 min; glycine was then added at room temperature for 5 min to quench the cross-linking. The cells were then collected in lysis buffer. The residual lysate was immunoprecipitated with normal rabbit IgG antibody. Chromatin was digested by micrococcal nuclease. Lysate (2%) was used as an input reference. This was followed by incubation with 3 μg of anti-FOXR2 antibody (Abcam) or normal rabbit IgG followed by immunoprecipitating with Protein G Agarose Beads during overnight incubation at 4 °C with gentle shaking. The DNA crosslink was reversed by 5 mol/L NaCl and Proteinase K at 65 °C for 2 h and then DNA was purified. Immunoprecipitated DNA was amplified by PCR using specific primers. Primers used for chromatin immunoprecipitation PCR are shown in Table 3.

**Table 3:** Primers used for ChIP experiments

Gene	Binding site or Control	Sequence (5′- > 3′)	Product size (bp)	Annealing temperature (°C)
ZO-1	PCR1	F:CCGGCAGGTTTGCGTG	102	58
		R:GGCTTCGCCACGTAACTTC		
	PCR2	F:AACGAGAGCAACGCTTCTGA	166	55
		R:GCCGATACCCGACAGTTGTT		
	PCR3	F:CCGCAGACACCAAAAATCCC	210	54
		R:ACGCATGTCATCATAGCTCCT		
occludin	PCR1	F: GAATTGGTCACCGAGGGAGG	107	59
		R:GACCTGGCTCTCGGTTCCT		
	PCR2	F: AAGGTTCCATCCGAAGCAGG	203	56
		R:CTGAAACTCGGATGGGAGGG		
	PCR3	F:GAGTTTTGTTCACTCGTGTGTGT	105	56
		R:TGTTGTGTTGGGTAGAAGGC		
claudin-5	PCR1	F: AGAAGGCAGTGGTCCTCAGA	185	62
		R: CTTCTCCCCCTTCAGATGGC		
	PCR2	F:CGAAGTAGGTGAGCAGACCC	204	56
		R:CACTATGTTGGTCGAGGCCC		

### Statistical analysis

Data are denoted as the mean ± standard deviation (SD). The experimental results were analyzed using a Student’s t-test or single-factor analysis of variance, and *p <* 0.05 was considered significant.

## Results

### piR-DQ590027 overexpression decreases the expressions of ZO-1, occludin, and claudin-5 and increases the permeability of glioma-conditioned normal BBB

**Fig. 1:**
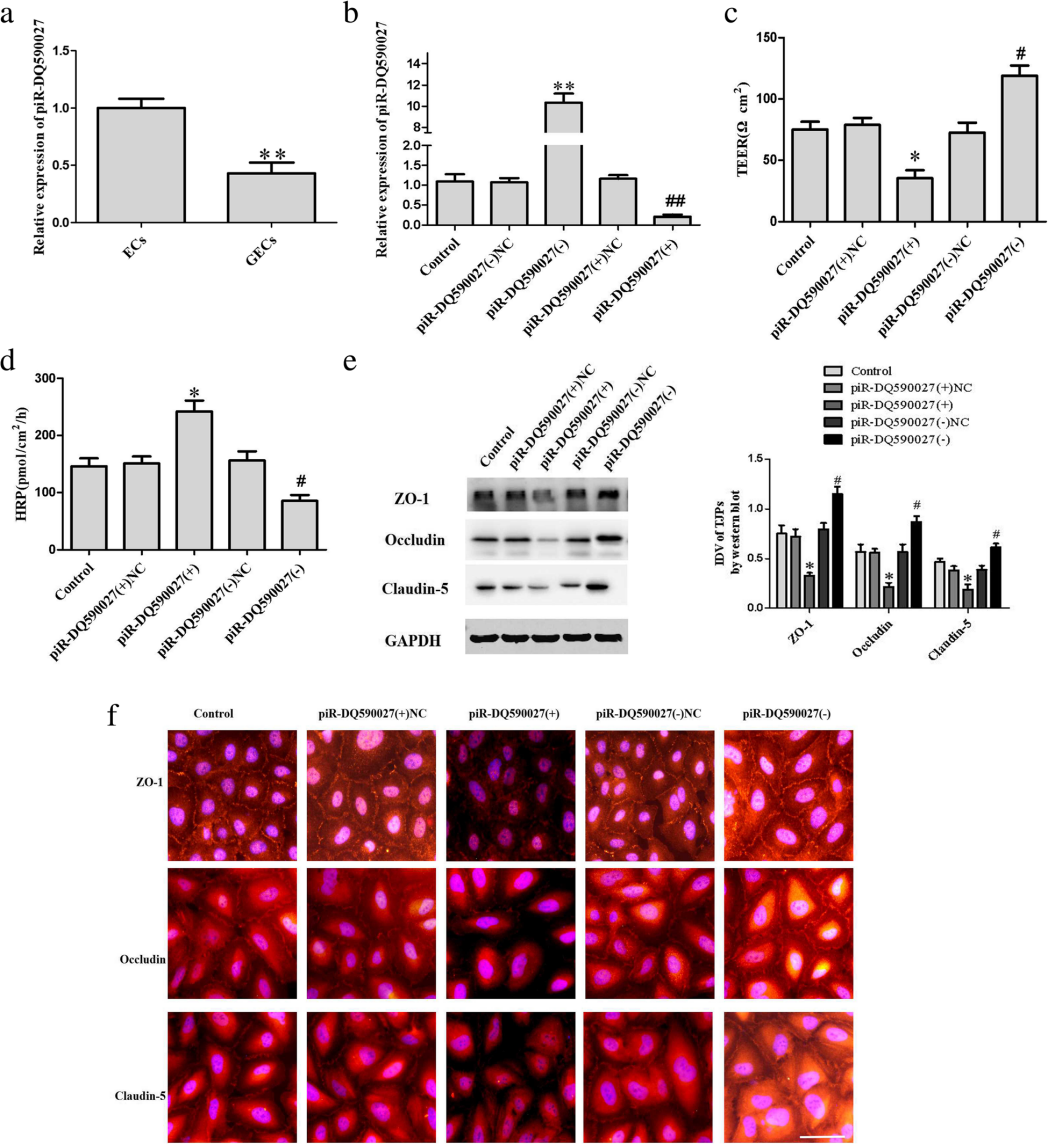
piR-DQ590027 increases the permeability of glioma-conditioned normal BBB and inhibits the expression of tight junction-related proteins. (**a**) Relative expression of piR-DQ590027 in ECs and GECs on quantitative real-time PCR. Data represent the mean ± SD (*n* = 3, each). **P < 0.01 vs. the EC group. (**b**) Transfection efficiency of piR-DQ590027 in GECs. (**c**) Effects of piR-DQ590027 overexpression and knockdown on TEER values in the in vitro glioma-conditioned normal BBB model. (**d**) Effects of piR-DQ590027 overexpression and knockdown on HRP flux in the in vitro glioma-conditioned normal BBB model. (**e**) Effects of piR-DQ590027 overexpression and knockdown on the expression of tight junction-related proteins on western blot. Data of B-E represent the mean ± SD (*n* = 3, each). *P < 0.05 vs. the piR-DQ590027 (+) NC group, **P < 0.01 vs. the piR-DQ590027 (+) NC group, ^#^*P* < 0.05 vs. the piR-DQ590027 (+) NC group, ^##^*P* < 0.01 vs. the piR-DQ590027 (+) NC group. (F) Effects of piR-DQ590027 overexpression and knockdown on the expressions and distributions of tight junction-related proteins on immunofluorescence (*n* = 3, each). Scale bar, 30 μm

We first determined the expression of piR-DQ590027 in ECs and glioma-conditioned ECs (GECs). As shown in Fig. 1a, its expression in GECs was significantly lower than ECs (*P <* 0.01). Overexpression and silencing of piR-DQ590027 were performed via transient transfection. The transfection efficacy is shown in Fig. 1b. Compared with the piR-DQ590027 (+) NC group, the TEER value of GECs in the piR-DQ590027 (+) group was decreased (*P* < 0.05) (Fig. 1c) and the HRP flux was increased (*P <* 0.05) (Fig. 1d). Meanwhile, compared with the piR-DQ590027 (−) NC group, the TEER value in the piR-DQ590027 (−) group was elevated (*P <* 0.05) (Fig. 1c) and the HRP flux was reduced (*P <* 0.05) (Fig. 1d). These findings suggest that piR-DQ590027 overexpression increases the permeability of glioma-conditioned normal BBB, in contrast to piR-DQ590027 silencing.

The effects of piR-DQ590027 overexpression on ZO-1, occludin, and claudin-5 are shown in Fig. 1e. piR-DQ590027 overexpression significantly decreased the expressions of ZO-1, occludin, and claudin-5 in GECs (*P* < 0.05). The expression and distribution of the above proteins were further determined by immunofluorescence (Fig. 1f). piR-DQ590027 overexpression weakened the immune response of ZO-1, occludin and claudin-5, these proteins exhibited a discontinuous distribution at the edge of GECs.

### Silencing of MIR17HG expression downregulates the expressions of ZO-1, occludin, and claudin-5 and increases the permeability of glioma-conditioned normal BBB

**Fig. 2:**
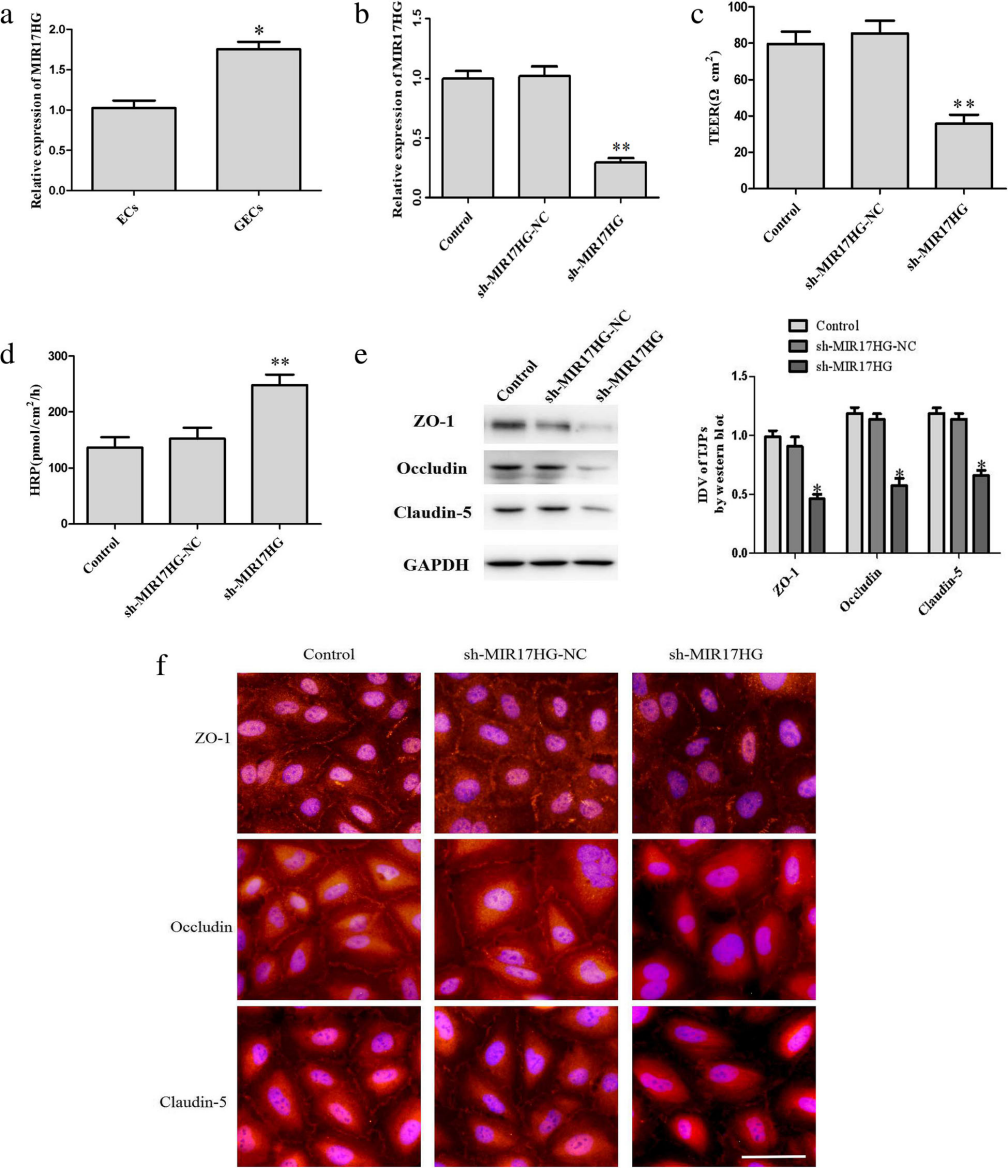
MIR17HG knockdown increases the permeability of glioma-conditioned normal BBB and decreases the expression of tight junction-related proteins. (**a**) Relative MIR17HG expression in ECs and GECs on quantitative real-time PCR. Data represent the mean ± SD (*n* = 3, each). *P < 0.05 vs. the EC group. (**b**) Transfection efficiency of shMIR17HG. (**c**) Effects of MIR17HG knockdown on TEER values in the in vitro glioma-conditioned normal BBB model. (**d**) Effects of MIR17HG knockdown on HRP flux in the in vitro glioma-conditioned normal BBB model. (**e**) Effects of MIR17HG knockdown on the expression of tight junction-related proteins on western blot. Data of B-E represent the mean ± SD (*n* =3, each). *P < 0.05 vs. the shMIR17HG NC group, **P < 0.01 vs. the shMIR17HG NC group. (**f**) Effects of MIR17HG knockdown on the expression of tight junction-related proteins and their distributions on immunofluorescence (*n* = 3, each). Scale bar, 30 μm

As shown in Fig. 2a, MIR17HG was expressed in both ECs and glioma-conditioned ECs (GECs). Compared with ECs, the endogenous expression of MIR17HG in GECs was significantly higher than ECs (*P <* 0.05). MIR17HG expression was then silenced by stable transfection with shMIR17HG. The transfection efficacy of shMIR17HG was shown in Fig. 2b. Compared with the shMIR17HG (−) NC group, the TEER value in the shMIR17HG group was decreased (*P <* 0.01) (Fig. 2c) and the HRP flux was increased (*P <* 0.01) (Fig. 2d). These findings suggest that silencing of MIR17HG expression increases the permeability of glioma-conditioned normal BBB.

Figure 2e shows the effects of silencing of MIR17HG expression on the expressions of ZO-1, occludin, and claudin-5. MIR17HG silencing clearly lowered the expression of the above three proteins in GECs (*P <* 0.05). The immunofluorescence results are shown in Fig. 2f. Silencing of MIR17HG weakened the immune response of ZO-1, occludin and claudin-5, these proteins exhibited a discontinuous distribution at the edge of GECs.

### piR-DQ590027 regulates the permeability of glioma-conditioned normal BBB by targeted-binding to MIR17HG

**Fig. 3:**
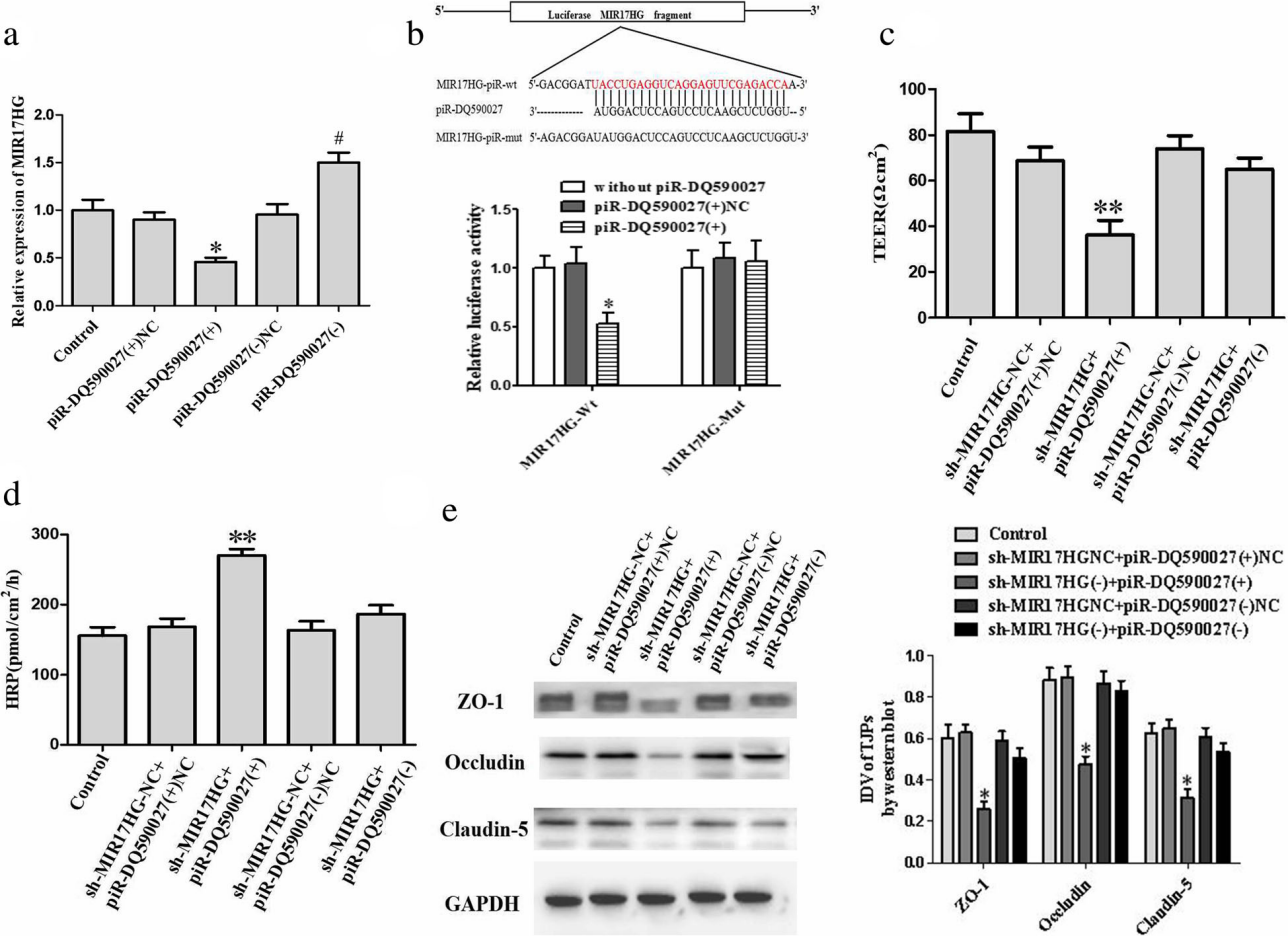
piR-DQ590027 negatively regulates MIR17HG and MIR17HG is involved in piR-DQ590027-induced changes of the permeability of glioma-conditioned normal BBB and expressions of tight junction-related proteins. (**a**) Relative MIR17HG expression levels in piR-DQ590027-overexpressing or knockdown GECs on quantitative real-time PCR. Data represent the mean ± SD (*n* = 3, each). *P < 0.05 vs. the piR-DQ590027 (+) NC group; ^#^*P* < 0.05 vs. the piR-DQ590027 (-) NC group. (**b**) Relative luciferase activity in HEK293T cells co-transfected with pmirGLO-MIR17HG-Wt (pmirGLO-MIR17HG-Mut) and agomir-piR-DQ590027. Data represent the mean ± SD (*n* = 3, each). *P < 0.05 vs. the MIR17HG-Mut+ piR-DQ590027 (+) group. (**c**) TEER value was measured to evaluate the effects of MIR17HG and piR-DQ590027 on glioma-conditioned normal BBB integrity. (**d**) HRP flux was measured to evaluate the effects of MIR17HG and piR-DQ590027 on the permeability of glioma-conditioned normal BBB. (**e**) Western blot was performed to evaluate the effects of MIR17HG and piR-DQ590027 on the expression of tight junction-related proteins. Data of **c**-**e** represent the mean ± SD (*n* = 3, each). **P < 0.01 vs. the shMIR17HG-NC+piR-DQ590027 (+) NC group

piRNA Bank and piRNA predictor bioinformatics software analysis predicted a binding site for piR-DQ590027 in the base sequence of MIR17HG (Additional file 1: Figure S2A). The further regulation of expression are shown in Fig. 3a. Compared with the piR-DQ590027 (+) NC group, MIR17HG expression in the piR-DQ590027 (+) group was significantly decreased in GECs (*P* < 0.05). Compared with the piR-DQ590027 (−) NC group, a marked increase in MIR17HG expression was observed in GECs in the piR-DQ590027 (−) group (*P <* 0.05). Dual-luciferase reporter gene assay showed that luciferase activity was decreased in the pmirGLO-MIR17HG-Wt + piR-DQ590027 (+) cotransfection group (*P* < 0.05), but no significant change was shown in the pmirGLO-MIR17HG-Mut + piR-DQ590027 (+) cotransfection group (Fig. 3b).

To further clarify the ability of piR-DQ590027 to negatively regulate the effect of MIR17HG on the permeability of glioma-conditioned normal BBB, the TEER and HRP assay were performed. Compared with the shMIR17HG (−) + piR-DQ590027 (+) NC group, the TEER value in the shMIR17HG (−) + piR-DQ590027 (+) group was clearly lower (*P* < 0.01) (Fig. 3c), whereas the HRP flux was significantly elevated (*P* < 0.01) (Fig. 3d). Compared with the shMIR17HG (−) + piR-DQ590027 (+) NC group, reduced protein expression of ZO-1, occludin, and claudin-5 in GECs was found in the shMIR17HG (−) + piR-DQ590027 (+) group (*P* < 0.01). However, no significant change of the above three proteins was observed in the shMIR17HG (−) + piR-DQ590027 (−) group (Fig. 3e). These findings indicated that piR-DQ590027 might influnce the permeability of glioma-conditioned normal BBB by regulating MIR17HG expression.

### miR-153 and miR-377 are lowly expressed in glioma-conditioned ECs (GECs) and miR-153 or miR-377 overexpression increases the permeability of glioma-conditioned normal BBB

**Fig. 4:**
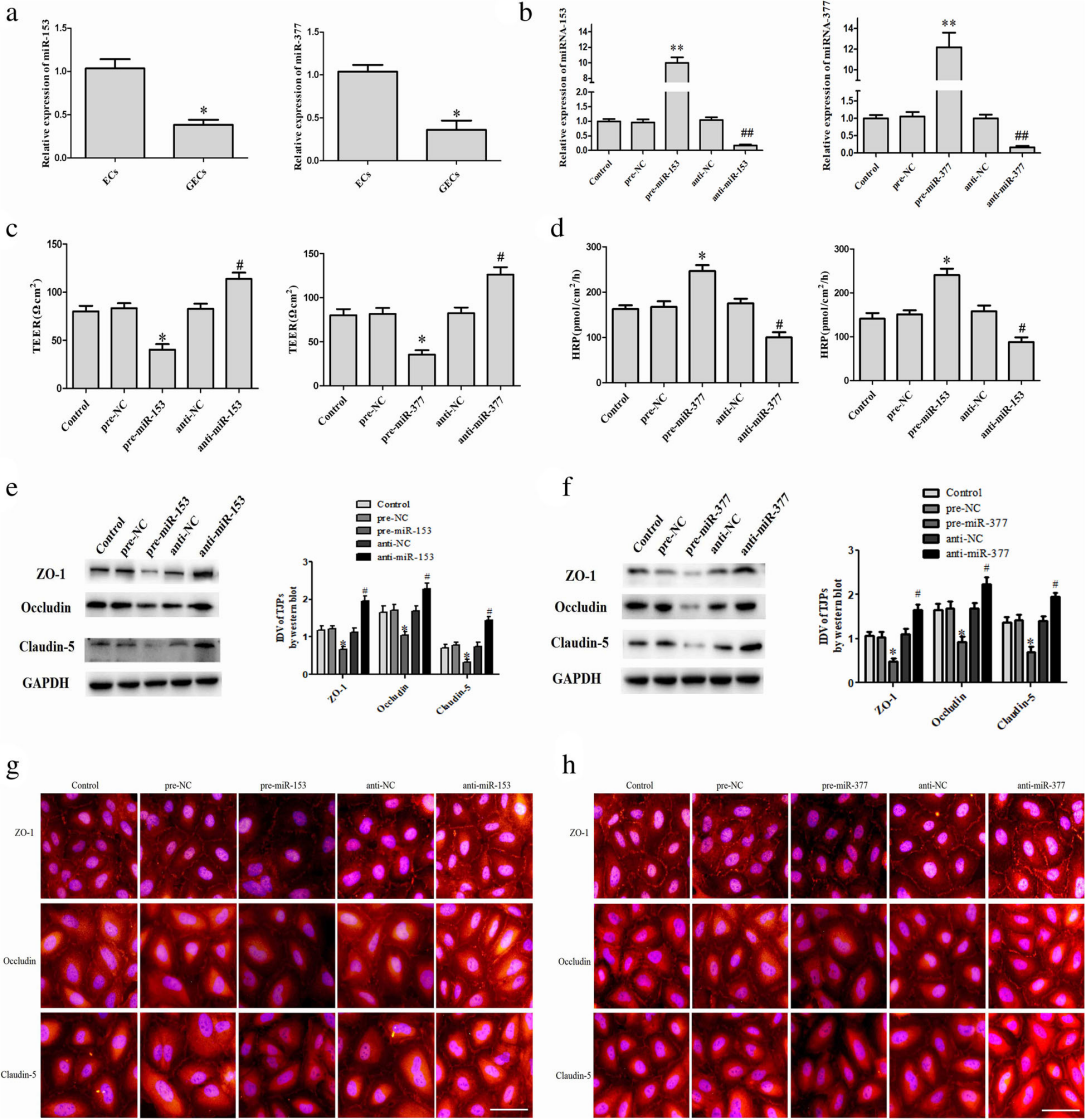
miR-153 and miR-377 overexpression increases the permeability of glioma-conditioned normal BBB and reduces tight junction-related protein expression. (**a**) miR-153 and miR-377 expression levels in ECs and GECs on quantitative real-time PCR. Data represent the mean ± SD (*n* = 3, each). *P < 0.05 vs. the EC group. (**b**) Transfection efficiency of miR-153 and miR-377 in GECs. (**c**) Effects of miR-153 and miR-377 on TEER values in the in vitro glioma-conditioned normal BBB model. (**d**) Effects of miR-153 and miR-377 on HRP flux in the in vitro glioma-conditioned normal BBB model. (**e**) Effects of miR-153 on the expression of tight junction-related proteins on western blot. (**f**) Effects of miR-153 on the expression of tight junction-related proteins on western blot. Data of **b**-**f** represent the mean ± SD (*n* = 3, each). *P < 0.05 vs. the pre-NC group, **P < 0.01 vs. the pre-NC group, ^#^*P* < 0.05 vs. the anti-NC group, ^##^*P* < 0.01 vs. the anti-NC group. (**g**) Effects of miR-153 on the expression of tight junction-related proteins and their distributions on immunofluorescence. Scale bar, 30 μm. (**h**) Effects of miR-377 on the expression of tight junction-related proteins and their distributions on immunofluorescence. Scale bar, 30 μm

As shown in Fig. 4a, miR-153 and miR-377 demonstrated significantly lower expression in GECs (*P* < 0.05). miR-153 and miR-377 were further overexpressed and silenced by transient transfection. The transfection efficacies are shown in Fig. 4b. After overexpression of miR-153 (pre-miR-153) and miR-377 (pre-miR-377) respectively, the TEER value was decreased (*P <* 0.05) and the HRP flux was increased (*P <* 0.05); after silencing of miR-153 and miR-377 respectively, there was a significant elevation in the TEER value (*P <* 0.05) (Fig. 4c) and a decline in the HRP flux (*P <* 0.05) (Fig. 4d). The effects of pre-miR-153 and pre-miR-377 on the expressions of ZO-1, occludin, and claudin-5 in GECs were shown in Fig. 4e and f. Compared with the pre-NC group, the protein expression of ZO-1, occludin, and claudin-5 was significantly decreased in both pre-miR-153 and pre-miR-377 groups (*P <* 0.05). Compared with the anti-NC group, their expression levels were significantly increased in both anti-miR-153 and anti-miR-377 groups (*P* < 0.05). The immunofluorescence results revealed that immune response of ZO-1, occludin, and claudin-5 weakened in the pre-miR-153 and pre-miR-377 groups, and these proteins exhibited a discontinuous distribution at the edge of GECs. However, in the anti-miR-153 and anti-miR-377 groups, the opposite result was observed (Fig. 4g and h).

### MIR17HG regulates the permeability of glioma-conditioned normal BBB by targeted-binding to miR-153 and miR-377

**Fig. 5:**
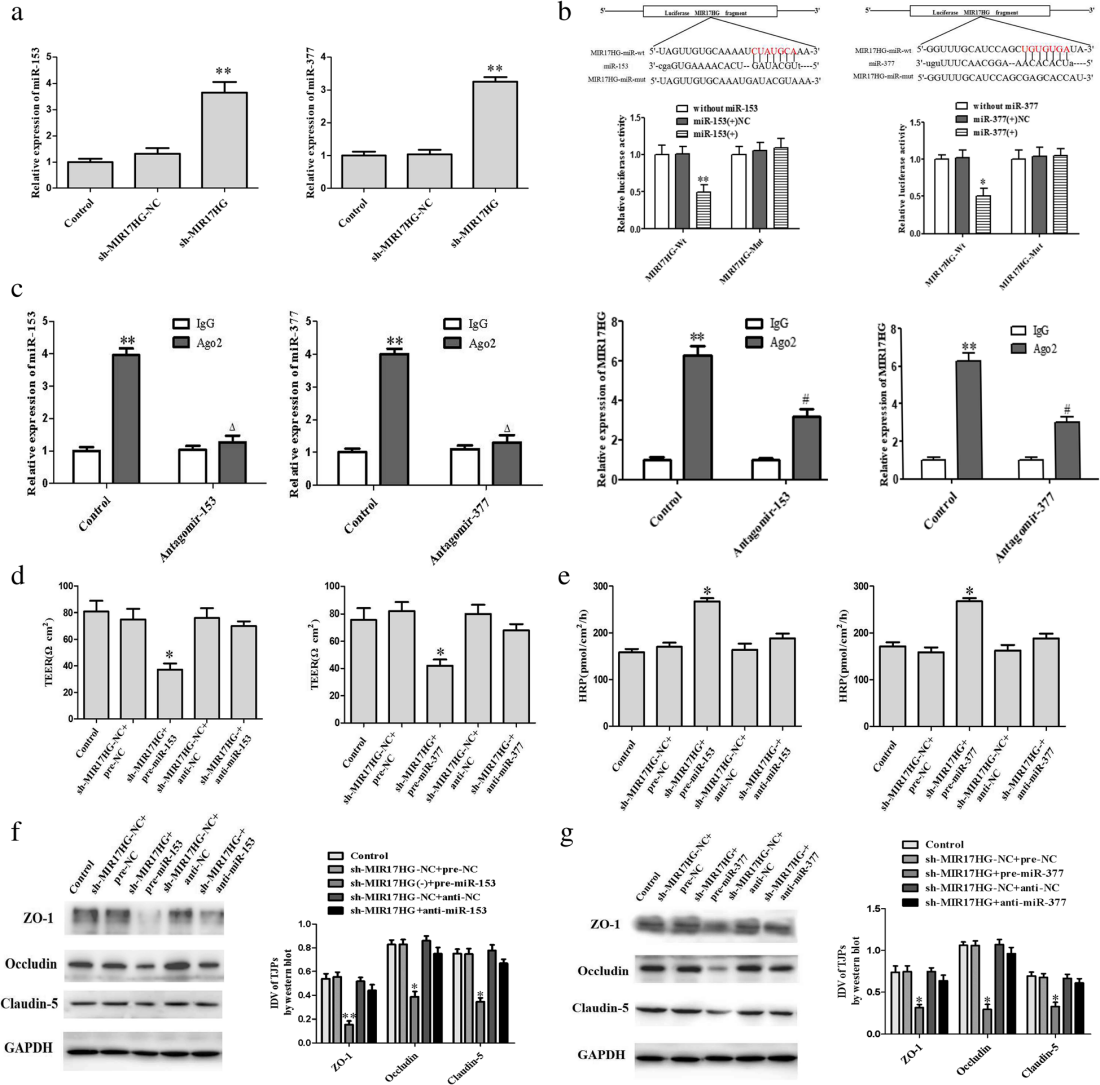
MIR17HG negatively regulates the eexpression of miR-153(mir-377) and miR-153(mir-377) is involved in MIR17HG-induced permeability regulation of glioma-conditioned normal BBB and tight junction-related proteins. (**a**) miR-153 (miR-377) expression levels in MIR17HG knockdown GECs on quantitative real-time PCR. Data represent the mean ± SD (*n* = 3, each). **P < 0.01 vs. shMIR17HG-NC groups. (**b**) Relative luciferase activity in HEK293T cells co-transfected with pmirGLO-MIR17HG-Wt (pmirGLO-MIR17HG-Mut) and agomir-153 (agomir-377). Data represent the mean ± SD (*n* = 3, each). **P < 0.01 vs. the MIR17HG-Wt + miR-153 (+) group, *P < 0.05 vs. the MIR17HG-Wt + miR-377 (+) group. (**c**) RNA immunoprecipitation assay performed with normal mouse IgG or anti-Ago2. Relative enrichment of MIR17HG and miR-153 (miR-377) was determined on quantitative real-time PCR. Data represent the mean ± SD (*n* = 3, each). **P < 0.01 vs. the IgG group, #*P* < 0.05 and △*P* < 0.05 vs. the IgG group. (**d**) TEER value was measured to evaluate the effects of MIR17HG and miR-153 (miR-377) on the permeability of glioma-conditioned normal BBB. (**e**) HRP flux was measured to evaluate the effects of MIR17HG and miR-153 (miR-377) on the permeability of glioma-conditioned normal BBB. (**f** and **g**) Western blot was performed to evaluate the effects of MIR17HG and miR-153 (miR-377) on tight junction-related proteins. Data of **d**-**g** represent the mean ± SD (*n* = 3, each). *P < 0.05 vs. the shMIR17HG-NC + pre-NC group, **P < 0.01 vs. the shMIR17HG-NC + pre-NC group

According to starBase 2.0 bioinformatics software (http://starbase.sysu.edu.cn/), MIR17HG was predicted to have separate binding sites for miR-153 and miR-377 (Additional file 1: Figure S2B). To validate whether MIR17HG could bind to miR-153 and miR-377 and regulate their expression, we first determined the expression of miR-153 and miR-377 in glioma-conditioned ECs (GECs) after silencing of MIR17HG (Fig. 5a). Compared with the shMIR17HG-NC group, the expression of miR-153 and miR-377 was significantly increased in the shMIR17HG group (*P <* 0.01). Further investigation was performed using a dual-luciferase reporter gene system and RNA pull-down assay. The results of dual-luciferase reporter gene showed that, compared with the pmirGLO-MIR17HG-Wt + miR-153 (+) NC cotransfection group, luciferase activity was decreased in the pmirGLO-MIR17HG-Wt + miR-153 (+) cotransfection group (*P <* 0.05). Compared with the pmirGLO-MIR17HG-Mut + miR-153 (+) NC cotransfection group, there was no significant change in the pmirGLO-MIR17HG-Mut + miR-153 (+) cotransfection group (Fig. 5b) or pmirGLO-MIR17HG-Mut + miR-377 (+) cotransfection group (Fig. 5b), but a marked decrease in the pmirGLO-MIR17HG-Wt + miR-377 (+) cotransfection group. The above findings indicated that MIR17HG bound to miR-153 and miR-377 in a sequence-specific manner.

The results of an RNA pull-down assay showed that, compared with the IgG coprecipitation group, the enrichment of both MIR17HG and miR-153 was significantly increased in the Ago2 coprecipitation group (*P <* 0.01) (Fig. 5c) and the enrichment of both MIR17HG and miR-377 was also enhanced (*P <* 0.01) (Fig. 5c); this assay indicated that MIR17HG, miR-153 and miR-377 are co-present in the Ago complex. Furthermore, we separately overexpressed and silenced miR-153 and miR-377 in GECs stably transfected with MIR17HG silencing and then determined the permeability of glioma-conditioned normal BBB. There was no significant difference between the control group and the shMIR17HG-NC + pre-NC group or shMIR17HG-NC + anti-NC group. Compared with the shMIR17HG-NC + pre-NC group, there was a significant decrease in the TEER value (*P <* 0.05) (Fig. 5d) and the expression of ZO-1 (*P <* 0.01), occludin, and claudin-5 (*P* < 0.05) (Fig. 5f and g) and an increase in HRP flux in the shMIR17HG + pre-miR-153 (pre-miR-377) group (*P <* 0.05) (Fig. 5e). For the TEER value, HRP flux (Fig. 5e), and expression of ZO-1, occludin, and claudin-5 (Fig. 5f and g), there were no significant differences between the shMIR17HG-NC + anti-NC and MIR17HG + anti-miR-153 (pre-miR-377) groups.

### FOXR2 separately binds to the promoter regions of ZO-1, occludin, and claudin-5 to promote their expression and regulate the permeability of glioma-conditioned normal BBB

**Fig. 6:**
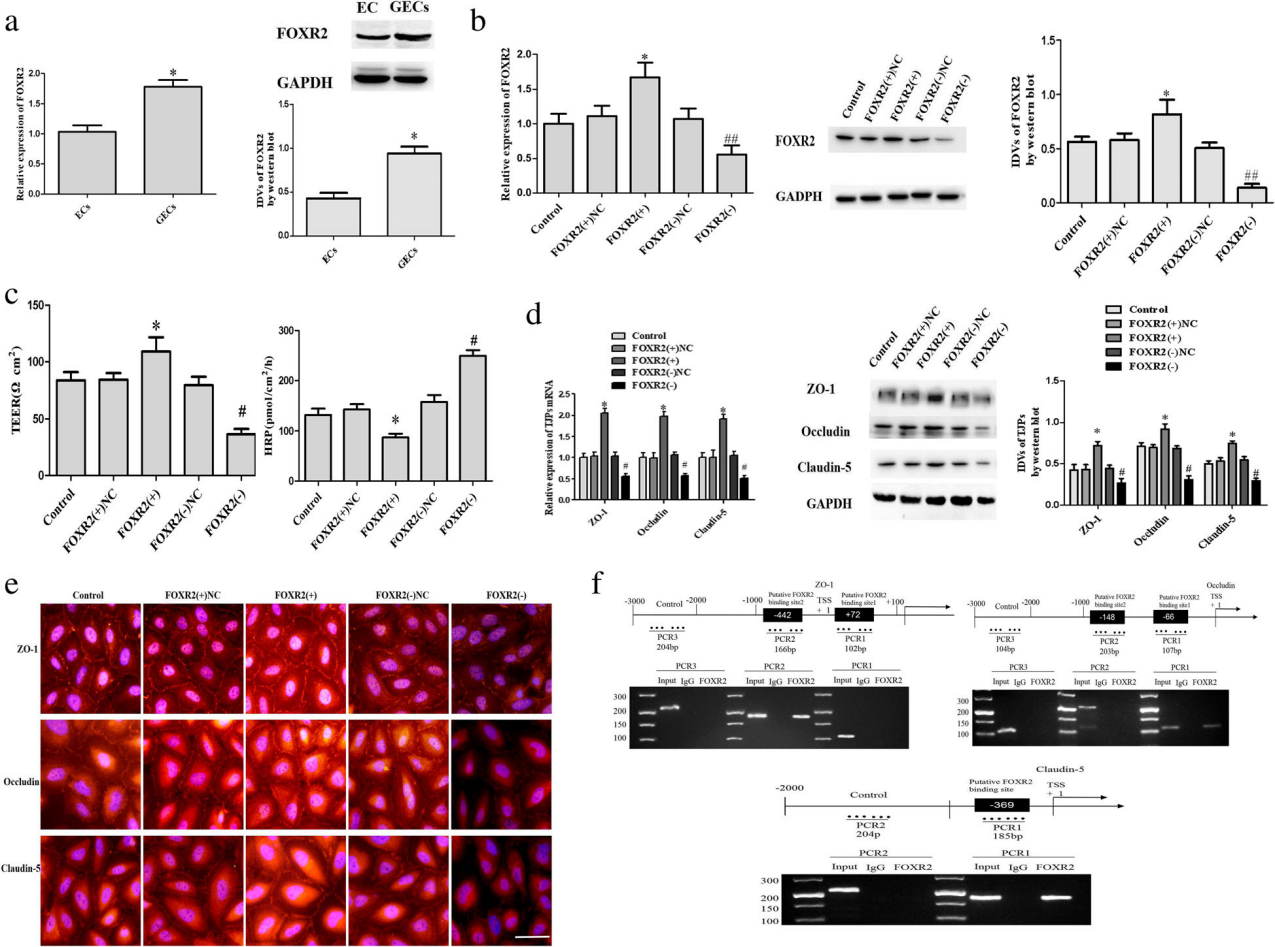
FOXR2 knockdown increases the permeability of glioma-conditioned normal BBB by decreasing the transcription of tight junction-related proteins. (**a**) FOXR2 expression levels in ECs and GECs on quantitative real-time PCR and western blot assay. Data represent the mean ± SD (*n* = 3, each). *P < 0.05 vs. EC groups. (**b**) Transfection efficiency of FOXR2 overexpression and knockdown on quantitative real-time PCR and western blot assay. (**c**) Effects of FOXR2 on TEER values and HRP flux in the in vitro glioma-conditioned normal BBB model. (**d**) Effects of FOXR2 on the mRNA and protein levels of tight junction-related proteins on quantitative real-time PCR and western blot assay. Data of **b**-**d** represent the mean ± SD (*n* = 3, each). *P < 0.05 vs. the FOXR2 (+) NC group, ^#^*P* < 0.05 vs. the FOXR2 (-) NC group; ^##^*P* < 0.05 vs. the FOXR2 (-) NC group. (**e**) Effects of FOXR2 on the expression of tight junction-related proteins and their distribution on immunofluorescence (*n* = 3, each). Scale bar, 30 μm. (**f**) FOXR2 bound to the promoter of ZO-1, occludin, and claudin-5 in GECs

With the help of TargetScan database, we predicted the binding sites of miR-153 and miR-377 in 3’UTR of FOXR2 (Additional file 1: Figure S2C). Then the endogenous expression of FOXR2 was detected and results showed mRNA and protein expression of FOXR2 in glioma-conditioned ECs (GECs) was significantly increased compared with in ECs (*P <* 0.05) (Fig. 6a). Subsequently, the stable transfected efficacy of FOXR2 in ECs after overexpression or silencing is shown in Fig. 6b TEER value and HRP flux in the glioma-conditioned normal BBB model were determined. Compared with the FOXR2 (+) NC group, the TEER value was significantly increased (*P <* 0.05) and the HRP flux was decreased (*P <* 0.05) in the FOXR2 (+) group; the protein expression of ZO-1, occludin, and claudin-5 was upregulated (*P <* 0.05). The results of the FOXR2 (−) group were opposite to those of the FOXR2 (+) group (Fig. 6c and d). The results of immunofluorescence showed the immune response of ZO-1, occludin, and claudin-5 enhanced in the FOXR2 (+) group and these proteins exhibited a continuous distribution at the edge of GECs. However, in the FOXR2 (−) groups, the immune response of ZO-1, occludin, and claudin-5 weakened in the FOXR2 (+) group and these proteins exhibited a discontinuous distribution at the edge of GECs.

FOXR subfamily proteins can bind to the FOXR core binding motif 5’-GACGC-3′ in the promoter region of target genes to further regulate the gene expression. We identified this “GACGC” sequence in the promoter regions of ZO-1, occludin, and claudin-5 in the DBTSS database (Additional file 1: Figure S2D). Then a chromatin co-immunoprecipitation test was conducted. We designed PCR primers downstream and upstream of the binding site and negative control PCR primers 1000 to 3000 bp upstream of the predicted FOXR2 transcription starting site of the above three genes. Based on the results (Fig. 6f), FOXR2 could bind to the predicted binding sites of ZO-1, occludin, and claudin-5, but no such binding was observed to the control regions.

### miR-153 and miR-377 bind to the 3’UTR of FOXR2 mRNA to inhibit FOXR2 expression and regulate the permeability of glioma-conditioned normal BBB

**Fig. 7:**
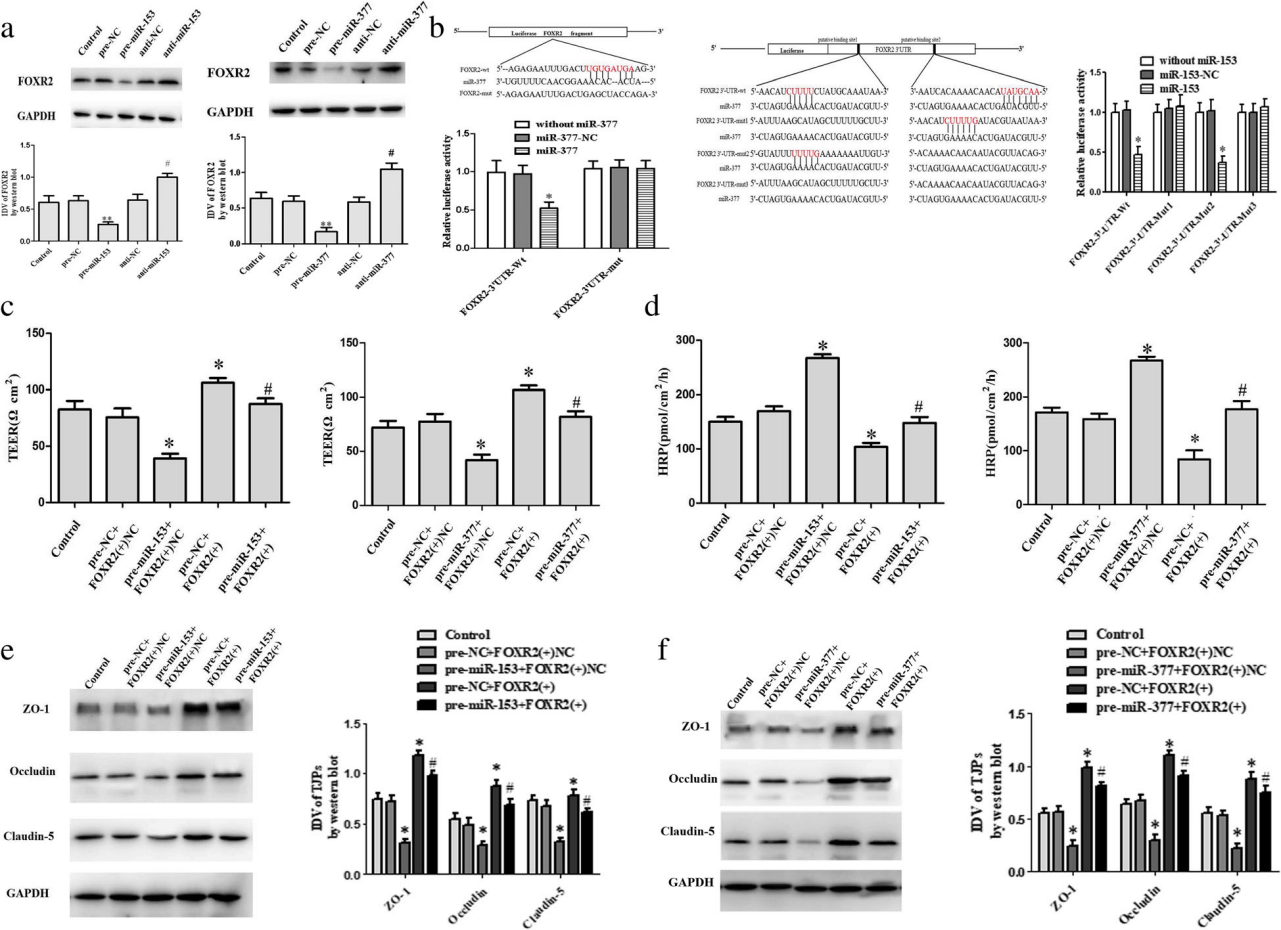
FOXR2 mediates the effects of miR-153 (miR-377) on the permeability of glioma-conditioned normal BBB. (**a**) Effects of miR-153 and miR-377 on FOXR2 expression. Data represent the mean ± SD (*n* = 3, each). **P < 0.01 vs. the pre-NC group, ^#^*P* < 0.05 vs. the anti-NC group. (**b**) Relative luciferase activity in HEK293T cells co-transfected with pmirGLO-FOXR2-Wt (pmirGLO-FOXR2-Mut) and agomir-377 (agomir-153). Data represent the mean ± SD (*n* = 3, each). *P < 0.05 vs. the FOXR2-3’-UTR-Wt + miR-377 (+) miR-153 (+) group. (**c**) TEER value was measured to evaluate the effects of FOXR2 and miR-153 (miR-377) on glioma-conditioned normal BBB integrity. (**d**) HRP flux was measured to evaluate the effects of FOXR2 and miR-153 (miR-377) on the permeability of glioma-conditioned normal BBB. (**e**) Western blot was performed to evaluate the effects of FOXR2 and miR-153 on tight junction-related proteins. (**f**) Western blot was performed to evaluate the effects of FOXR2 and miR-377 on tight junction-related proteins. Data of C-F represent the mean ± SD (*n* = 3, each). *P < 0.05 vs. the pre-NC + FOXR2 (+) NC group, ^#^*P* < 0.05 vs. the pre-miR-153 (pre-miR-377) + FOXR2 (+) NC group

Finally, the effects of miR-153 and miR-377 on FOXR2 expression were determined. Compared with the pre-NC group, FOXR2 expression was significantly downregulated in pre-miR-153 and pre-miR-377 groups (*P <* 0.01), but upregulated in anti-miR-153 and anti-miR-377 groups (*P <* 0.05) (Fig. 7a). The results of dual-luciferase reporter gene demonstrated the activity of luciferase was lower in the FOXR2–3’UTR-Wt + mmiR-377 (+) cotransfection group than in the FOXR2–3’UTR-Wt + miR-377 (+) NC cotransfection group (*P <* 0.05) (Fig. 7b). Compared with the FOXR2–3’UTR-Mut + miR-377 (+) NC cotransfection group, there was no significant change in the FOXR2–3’UTR-Mut + miR-377 (+) cotransfection group. In HEK293T cells, miR-153 effectively reduced the relative luciferase activity of the FOXR2 3′-UTR-Wt conduct and FOXR2 3′-UTR-Mut2, but not that of the FOXR2 3′-UTR-Mut1 and FOXR2 3′-UTR-Mut3 conducts (Fig. 7b), indicating that theputative binding site 1 was functional.Further investigation suggested that dual overexpression of miR-153 and FOXR2 significantly reversed the effects of FOXR2, as shown by increasing TEER value (*P* < 0.05) and the expressions of ZO-1, occludin, and claudin-5 (*P* < 0.05) and decreasing HRP flux (*P* < 0.05) (Fig. 7c–f); dual overexpression of miR-377 and FOXR2 had the similar effects (*P* < 0.05) (Fig. 7c–f).

## Discussion

We know that the permeability of glioma-conditioned normal BBB was increased (Additional file 1: Figure S1). This study showed that piR-DQ590027 was lowly expressed in glioma-conditioned ECs (GECs) and that piR-DQ590027 overexpression could decrease the expressions of ZO-1, occludin, and claudin-5 to further increase the permeability of glioma-conditioned normal BBB. However, piR-DQ590027 silencing increased the expressions of ZO-1, occludin, and claudin-5 and thus reduced the permeability of glioma-conditioned normal BBB. piRNAs might have several biological functions, including regulation of gene expression. hsa_piR_020365 and hsa_piR_008624 are highly expressed in glioma patients and negatively correlated with their prognosis [27]. A piR-598 allelic variation at rs147061479 increases the proliferation and survival of glioma cells [28]. Additionally, piR-DQ593109 is upregulated in GECs and piR-DQ593109 knockdown increases BTB permeability [29]. piRNA-DQ590027 has not been found in gliomas. This study showed for the first time that piR-DQ590027 was expressed at low levels in GECs. Therefore, we believe that piRNA-DQ590027 may play an inhibitory role in gliomas. The permeability of BBB/BTB is mostly increased via the transcellular pathway of caveolin-mediated transcytosis and the paracellular pathway mediated by tight junction opening [30–33]. Our study showed that piR-DQ590027 can decrease the expression of ZO-1, claudin-5, and occludin and increase the permeability of glioma-conditioned normal BBB, suggesting that piR-DQ590027 overexpression increases the permeability of glioma-conditioned normal BBB via the paracellular pathway. piRNA usually binds PIWI to form the PIWI–piRNA complex and then participates in such biological processes as mRNA degradation and translation regulation [34]. The specific PIWI proteins bound by piR-DQ590027 to increase the permeability of glioma-conditioned normal BBB need to be studied.

Increasing numbers of studies have shown that lncRNA plays an important role in regulating the permeability of BBB/BTB. The MIR17HG gene locus is located on chromosome 13q31.3 and encodes the miR-17-92 gene cluster (MIR17HG); the family members of this gene cluster are widely involved in the functional regulation of vascular ECs [35, 36]. miR-17-92 cluster amplification was also reported in neuroblastomas and is linked to poor prognosis [37]. Lastly, miR-17 promotes the growth of neuroblastoma cell lines [38]. Thus, we believe that MIR17HG is likely to play a cancer-promoting role in gliomas. We found that MIR17HG was highly expressed in GECs and MIR17HG silencing decreased the expressions of ZO-1, occludin, and claudin-5 and increased the permeability of glioma-conditioned normal BBB. This suggests that MIR17HG silencing regulates the permeability of glioma-conditioned normal BBB via the paracellular pathway. In the present study, the binding sites between MIR17HG and piR-DQ590027 were predicted with the help of bioinformatics software, and further results verified that MIR17HG was a downstream target of piR-DQ590027. In GECs, piR-DQ590027 overexpression significantly reduced the expression of MIR17HG, whereas piR-DQ590027 silencing clearly increased it, suggesting that piR-DQ590027 can negatively regulate the expression of MIR17HG. piR-DQ590027 overexpression and MIR17HG silencing markedly increased the permeability of glioma-conditioned normal BBB. The results indicate that piR-DQ590027 overexpression increases the permeability of glioma-conditioned normal BBB via the paracellular pathway by reducing the expressions of ZO-1, occludin, and claudin-5 in a manner that is related to the negative regulation of MIR17HG expression.

miR-153 and miR-377 play important regulatory roles in the occurrence and development of glioma. In glioma, miR-153 acts as an anti-oncogene via targeted-regulation of mTORC2 or DNA methylation [14, 39]. SNHG15 affects the growth of glioma microvascular ECs by negatively regulating miR-153 [40]. In addition, miR-153 is a tumor suppressor in glioblastoma stem cells [41]. Additionally, miR-153 overexpression decreases the radioresistance and stemness of glioma stem cells by targeting the Nrf-2/GPx1/ROS pathway [42]. At present, there are no reports in the literature on the regulatory role of miR-153 in the function of ECs. miR-377 had lower expression in pancreatic cancer tissues and cells and miR-377 overexpression can inhibit the proliferation and induce the apoptosis of pancreatic cancer cells [43]. In glioma, miR-377 suppresses the proliferation and invasion of tumor cells via targeted-regulation of SP1 [19]. No other role has been reported in gliomas, and we suspect that it is likely to act similarly to miR-153, which can inhibit gliomas. There are no reports on the effects of miR-377 on vascular ECs. miR-377 can prevent ischemic brain injury, and miR-377 silencing promotes the proliferation, migration, and tubularization of cerebral microvascular ECs [44]. Our results showed that both miR-153 and miR-377 had lower expression in GECs. Overexpression of miR-153 or miR-377 reduced the expressions of ZO-1, occludin, and claudin-5 and further increased the permeability of glioma-conditioned normal BBB. In contrast, silencing of miR-153 or miR-377 increased the expressions of ZO-1, occludin, and claudin-5 and decreased the permeability of glioma-conditioned normal BBB. Based on software prediction of binding sites for miR-153 and miR-377 in MIR17HG, we further proved that miR-153 and miR-377 were downstream target genes of MIR17HG. MIR17HG silencing increased the expressions of both miR-153 and miR-377 in GECs. MIR17HG silencing and overexpression of miR-153 or miR-377 significantly decreased the expressions of ZO-1, occludin, and claudin-5 and increased the permeability of glioma-conditioned normal BBB; however, the dual silencing of both MIR17HG + miR-153 and MIR17HG + miR-377 had no significant effects on the permeability of glioma-conditioned normal BBB. This indicates that MIR17HG silencing increases the permeability of glioma-conditioned normal BBB by negatively regulating the expression of miR-153 and miR-377.

FOXR2 is a member of the forkhead box transcription factor family and a DNA-binding transcription factor. FOXR2 is highly expressed in medulloblastoma and acts as a carcinogen [26]. The present study demonstrated that FOXR2 had high expression in GECs, and silencing of FOXR2 decreased the expressions of ZO-1, occludin, and claudin-5 and increased the permeability of glioma-conditioned normal BBB. However, FOXR2 overexpression had the opposite effect. Based on bioinformatics software prediction that FOXR2 had binding sites for miR-153 and miR-377, our findingd showed that miR-153 and miR-377 bound to 3’UTR of FOXR2 respectively, which confirmed that FOXR2 was a target gene of miR-153 and miR-377. Overexpression of miR-153 or miR-377 significantly reduced the protein expression of FOXR2, whereas silencing of miR-153 or miR-377 clearly increased it. Meanwhile, the co-transcription experimental results of miR-153 (miR-377) and FOXR2 proved that miR-153 (miR-377) overexpression increased the permeability of glioma-conditioned normal BBB, whereas FOXR2 overexpression decreased it; the dual overexpression of miR-153 (miR-377) and FOXR2 reversed the effects of overexpressed miR-153 (miR-377) alone in increasing the permeability of glioma-conditioned normal BBB and decreasing the expressions of ZO-1, occludin, and claudin-5. The above results indicate that miR-153 and miR-377 affect the permeability of glioma-conditioned normal BBB by targeting FOXR2 and upregulate the expression of FOXR2 at the post-transcriptional level to further regulate the expressions of ZO-1, occludin, and claudin-5 and influence the permeability of glioma-conditioned normal BBB.

miRNAs play important roles in regulating the permeability of BBB/BTB. miR-200b regulates RMP-7 to increase the permeability of BTB by negatively modulating the target genes RhoA and ROCKII [45]. miR-18a overexpression binds to MEF2D and negatively regulates its expression; MEF2D then binds to the promoter region of KLF4, and finally KLF4 decreases the expressions of ZO-1, occludin, and claudin-5 and increases the permeability of BTB [46]. Overexpressed miR-18a also binds to RUNX1 and then negatively regulates its expression, and then the decrease in RUNX1 leads to inhibition of the transcription and thus the expressions of ZO-1, occludin, and claudin-5, further increasing BTB permeability [47]. miR-34c regulates the transcription and then influences the expressions of ZO-1, occludin, and claudin-5 by targeted-regulation of the transcription factor MAZ and finally controls the permeability BTB [48].

The transcription factor FOXR2 can directly bind to promoters to regulate gene transcription. Affected genes include CCNA1, CCND1, XRCC4, XRCC6, p15, and COL1A1 in MDA-MB-468 cells [23]. By analyzing the promoter sequence, we found evidence of potential binding between FOXR2 and the promoter regions of ZO-1, occludin, and claudin-5. Silencing of FOXR2 expression significantly decreased the mRNA and protein expression of ZO-1, occludin, and claudin-5, reduced their distribution in the cell membrane, and increased the permeability of glioma-conditioned normal BBB; FOXR2 overexpression had the opposite effects. The above results suggest that the piR-DQ590027/MIR17HG/miR-153 (miR-377)/FOXR2 pathway plays an important role in the regulation of the permeability of glioma-conditioned normal BBB.

Taken together, this study shows (Additional file 1: Figure S3) that piR-DQ590027, miR-153, and miR-377 are lowly expressed in GECs, whereas MIR17HG and FOXR2 are highly expressed. piR-DQ590027 overexpression reduces the expression of MIR17HG. Upregulation of MIR17HG enhances the post-transcriptional inhibition of FOXR2 induced by miR153 and miR-377. Moreover, downregulation of FOXR2 decreases the transcription and thus the expressions of ZO-1, occludin, and claudin-5, and thereby increases the permeability of glioma-conditioned normal BBB. Our study results, from the view of non-coding RNA regulation of BBB permeability, show that the piR-DQ590027/MIR17HG/miR-153 (miR-377)/FOXR2 pathway plays an important role in regulating the permeability of glioma-conditioned normal BBB. This study provides a new molecular mechanism for the open regulation of the BBB and a new potential avenue for glioma treatment.

## Conclusions

Taken together, this study firstly demonstrated that piR-DQ590027, miR-153 and miR-377 in GECs had lower expression, while MIR17HG and FOXR2 had higher expression. Overexpression of piR-DQ590027 could reduce the expression of MIR17HG. Furthermore, up-regulation of MIR17HG enhanced the post-transcriptional inhibition in FOXR2 induced by miR153 and miR-377. Moreover, down-regulation of FOXR2 inhibited transcription and decreased the expressions of ZO-1, occludin and claudin-5, and further increased the permeability of glioma-conditioned normal BBB. Our findings, from a view of non-coding RNA regulating the permeability of BBB/BTB, prove that piR-DQ590027/MIR17HG/miR-153(miR-377)/FOXR2 pathway plays an important role in regulating the permeability of glioma-conditioned normal BBB. This study provides a new molecular mechanism for the open regulation of BBB and a new idea for glioma treatment.

## Additional file

Additional file 1: **Figure S1.** The permeability of glioma-conditioned normal BBB was increased. “ECs” represents co-cultured ECs (hCMEC/D3) with human astrocytes; “GECs” represents co-cultured ECs (hCMEC/D3) with glioma cells. (A) TEER values in the in vitro glioma-conditioned normal BBB model. Data represent the mean ± SD (*n* = 3, each). *P < 0.05 vs. the ECs group. (B) HRP flux in the in vitro glioma-conditioned normal BBB model. Data represent the mean ± SD (*n* = 3, each). *P < 0.05 vs. the ECs group. **Figure S2.** The prediction results of Bioinformatics software. (A) The predicted binding site for piR-DQ590027 in the base sequence of MIR17HG. (B) The predicted binding sites of MIR17HG and miR-153/miR-377. (C) The predicted binding sites of miR-153 and miR-377 in 3’UTR of FOXR2. (D) The predicted binding sites of FOXR2 and the promoter regions of ZO-1, claudin-5 and occludin respectively (“yeloow” represents the binding sites; “red” represents transcription start site). **Figure S3.** The schematic cartoon underlying the mechanism of piR-DQ590027/MIR17HG regulating the permeability of glioma conditioned normal BBB. (PDF 861 kb)

## Abbreviations

BBB: Blood-brain barrier
BTB: Blood-tumor barrier
ECs: Endothelial cells (Endothelial cells co-culturing with Human astrocytes cells)
GECs: Glioma-conditioned ECs (Endothelial cells co-culturing with U87 cells)
lncRNA: Long non-coding RNA
piRNA: PIWI-associated RNAs
