# Supplementary material for: Mechanism of piR-DQ590027/MIR17HG regulating the permeability of glioma conditioned normal BBB
Source: J Exp Clin Cancer Res. 2018 Oct 11;37:246. doi: 10.1186/s13046-018-0886-0 (PMC6180493; doi:10.1186/s13046-018-0886-0)
Supplement: Supplementary file 1 — Figure S1. The permeability of glioma-conditioned normal BBB was increased. “ECs” represents co-cultured ECs (hCMEC/D3) with human astrocytes; “GECs” represents co-cultured ECs (hCMEC/D3) with glioma cells. (A) TEER values in the in vitro glioma-conditioned normal BBB model. Data represent the mean ± SD (n = 3, each). *P < 0.05 vs. the ECs group. (B) HRP flux in the in vitro glioma-conditioned normal BBB model. Data represent the mean ± SD (n = 3, each). *P < 0.05 vs. the ECs group. Figure S2. The prediction results of Bioinformatics software. (A) The predicted binding site for piR-DQ590027 in the base sequence of MIR17HG. (B) The predicted binding sites of MIR17HG and miR-153/miR-377. (C) The predicted binding sites of miR-153 and miR-377 in 3’UTR of FOXR2. (D) The predicted binding sites of FOXR2 and the promoter regions of ZO-1, claudin-5 and occludin respectively (“yeloow” represents the binding sites; “red” represents transcription start site). Figure S3. The schematic cartoon underlying the mechanism of piR-DQ590027/MIR17HG regulating the permeability of glioma conditioned normal BBB. (PDF 861 kb) [file 13046_2018_886_MOESM1_ESM.pdf]

Additional file

Title: Mechanism of piR-DQ590027/MIR17HG Regulation of Permeability across the glioma conditioned normal BBB

Additional file Summary

-- Figure Legends

- Figures

Figure.S1 The permeability of glioma-conditioned normal BBB was increased. "ECs" represents co-cultured ECs (hCMEC/D3) with human astrocytes; "GECs" represents co-cultured ECs (hCMEC/D3) with glioma cells. (A) TEER values in the *in vitro* glioma-conditioned normal BBB model. Data represent the mean ± SD (n = 5, each). *P < 0.05 vs. the ECs group. (B) HRP flux in the *in vitro* glioma-conditioned normal BBB model. Data represent the mean ± SD (n=5, each). *P<0.05 vs. the ECs group.

Figure.S2 The prediction results of Bioinformatics software. (A) The predicted binding site for piR-DQ590027 in the base sequence of MIR17HG. (B) The predicted binding sites of MIR17HG and miR-153/miR-377. (C) The predicted binding sites of miR-153 and miR-377 in 3'UTR of FOXR2. (D) The predicted binding sites of FOXR2 and the promoter regions of ZO-1, claudin-5 and occludin respectively ("yeloow" represents the binding sites; "red" represents transcription start site).

Figure.S3 The schematic cartoon underlying the mechanism of piR-DQ590027 /MIR17HG regulating the permeability of glioma conditioned normal BBB.

Figure S1

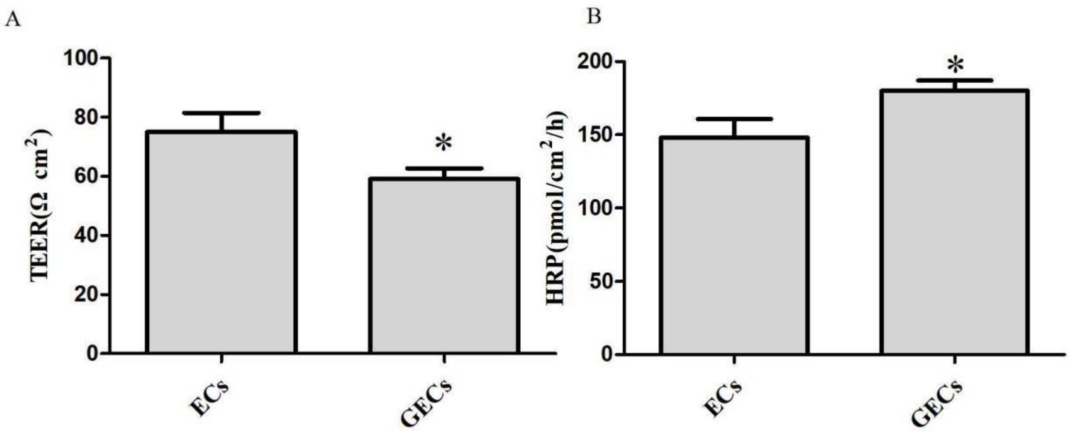

Figure S2

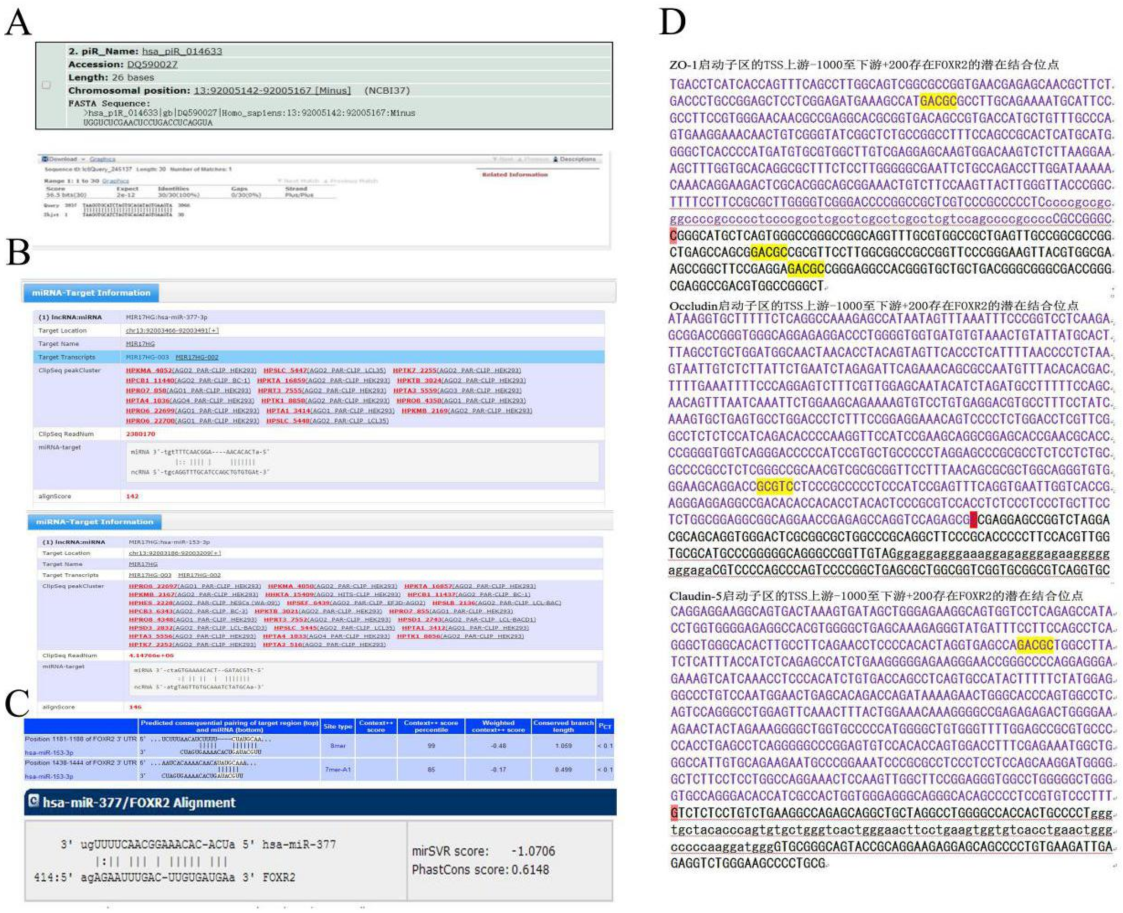

Figure S3

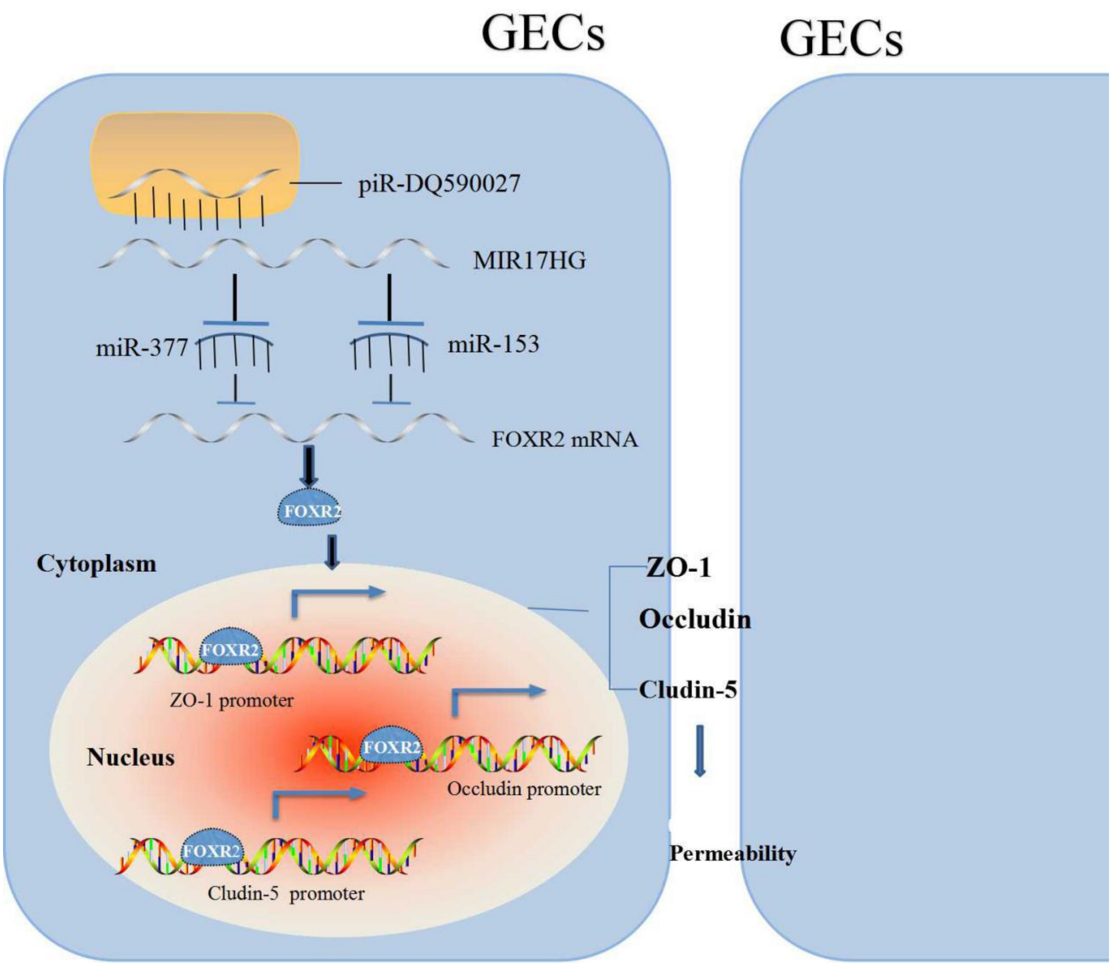
GECs
GECs
piR-DQ590027
MIR17HG
miR-377
miR-153
FOXR2 mRNA
FOXR2
Cytoplasm
ZO-1
Occludin
Cludin-5
FOXR2
ZO-1 promoter
Nucleus
FOXR2
Occludin promoter
FOXR2
Cludin-5 promoter
Permeability
